# Acute Stroke in Middle Cerebellar Peduncle in a Patient With FXTAS

**DOI:** 10.3389/fgene.2018.00187

**Published:** 2018-05-25

**Authors:** Deborah A. Hall, Avram Fraint, Rima Dafer

**Affiliations:** Department of Neurological Sciences, Rush University Medical Center, Chicago, IL, United States

**Keywords:** FXTAS, MCP, middle cerebellar peduncle, Fragile X, FXS, stroke

## Abstract

**Background:** Fragile-X associated tremor/ataxia syndrome (FXTAS) is commonly associated with T2 hyperintensity in the middle cerebellar peduncles (MCP) on magnetic resonance imaging (MRI). However, ischemic stroke in the MCP in a patient with FXTAS has not previously been described.

**Case Description:** A 61-year-old man with hypertension, sleep apnea, obesity, and FXTAS presented to the emergency department with 2 days of worsening balance and nausea which began 2 days after chiropractic neck manipulation. Examination revealed new nystagmus and worsening dysmetria. Workup revealed an acute infarct in the left MCP, atherosclerotic narrowing of the V4 segment of the left vertebral artery, inadequately controlled hypertension, and a LDL of 127.

**Conclusion:** Isolated MCP infarcts are rare and typically associated with hypoperfusion in the setting of vertebral artery disease and neck manipulation. We suspect that underlying neurodegeneration due to FXTAS with superimposed small vessel disease and neck manipulation may have caused preferential damage to the Purkinje cells in the MCP.

## Introduction

A 61-year-old man with obesity, hypertension, and obstructive sleep apnea presented initially with numbness and tingling in his left hand and heaviness in his legs. At that time he also endorsed balance issues – noting that he would veer to the right and stumble when he was overly tired. He reported three falls in the few years before presentation. He also endorsed movements of his extremities and talking during sleep, memory decline, anxiety, depression, and a personality change described as increased apathy. He had a grandson with Fragile X syndrome and two daughters who were premutation carriers (56 and 62 *FMR1* CGG repeats). He had a niece who had developed primary ovarian insufficiency and menopause at age 28.

Initial physical exam was notable for saccadic pursuits and hypometric saccades on testing of extraocular movements. His speech was mildly dysarthric. He had increased tone bilaterally, mild bilateral rest tremor, mild bradykinesia in his right upper extremity, and bilateral action tremor when drawing spirals. Sensory exam was notable for decreased vibratory and pinprick sensation in both feet. He was unable to stand on one foot and had difficulty with tandem gait. Genetic testing showed 54 *FMR1* CGG repeats. Magnetic resonance imaging (MRI) of his brain obtained at the time of this initial evaluation was notable for extensive T2 hyperintense signal throughout the subcortical and periventricular white matter in the frontal and parietal lobes as well as periventricular white matter hyperintensities in the occipital and temporal lobes. There were no MRI changes in the middle cerebellar peduncle (MCP) or corpus callosum. Based on the combination of his physical exam findings and his genetic testing, a diagnosis of definite Fragile X-associated tremor/ataxia syndrome (FXTAS) was made.

Six months later he presented to the emergency department (ED) with 2 days of worsening gait imbalance. He described a visit to his chiropractor 4 days before presentation for a “re-alignment” of his neck and back. Two days after the visit, he had an acute deterioration in his gait and balance as well as multiple episodes of vomiting. He waited two more days before coming into the ED for evaluation.

**FIGURE 1:**
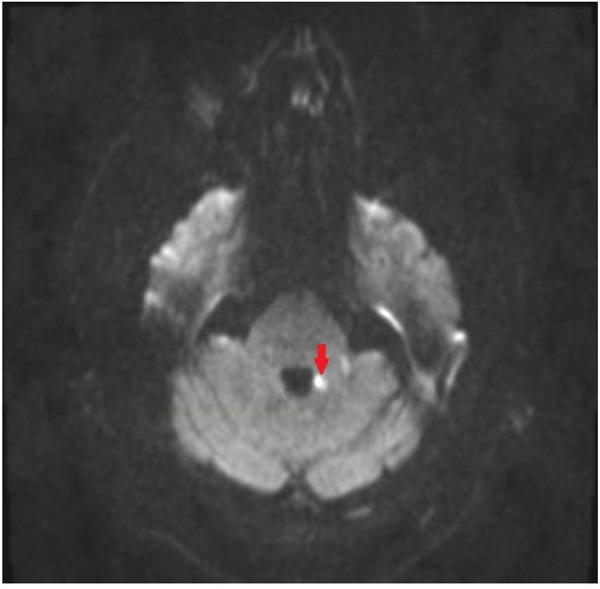
Magnetic resonance imaging (MRI) of brain of patient. Diffusion weighted image showing restricted diffusion in the medial aspect of the left middle cerebellar peduncle.

**FIGURE 2:**
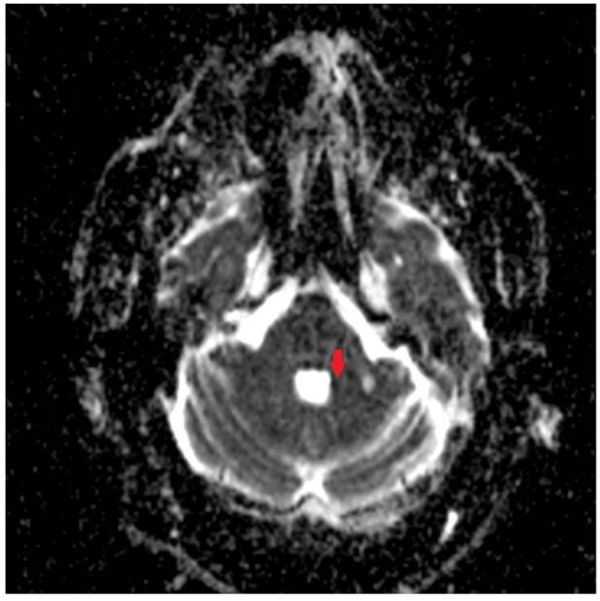
Apparent Diffusion Coefficient (ADC) image showing corresponding acute ischemic change in the medial aspect of the left middle cerebellar peduncle.

**FIGURE 3:**
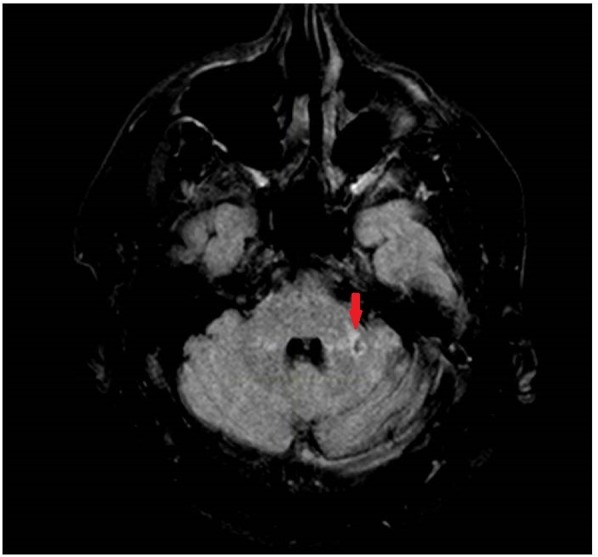
T2 Fluid Attenuated Inversion Recovery (FLAIR) image showing a chronic infarct in the lateral aspect in the left middle cerebellar peduncle.

Examination in the ED revealed new gaze-evoked nystagmus on horizontal gaze which was sustained when looking to the left. His tremor, tone, bradykinesia, sensory examination, and reflexes were unchanged. He also had new dysmetria on bilateral finger-to-nose testing, had a slow, cautious, wide-based gait, and was now unable to stand in tandem. CT angiography of his head and neck vessels was ordered given concern for a dissection, and these were negative. MRI of his brain showed an acute punctate infarct in the medial aspect of the left MCP (**Figures 1**, **2**) as well as a new chronic infarct in the lateral aspect of the left MCP (**Figure 3**). MRA revealed atherosclerotic narrowing of the left V4 segment of the vertebral artery. The remaining stroke workup revealed an LDL of 127 and inadequately controlled hypertension.

## Background

Fragile X-associated tremor/ataxia syndrome (FXTAS) is a movement disorder caused by expansion in the trinucleotide CGG repeat in the promoter region in the *fragile X mental retardation 1 (FMR1)* gene. The prevalence of the *FMR1* mutation is 1/151–1/209 in women and 1/468–1/430 in men (Seltzer et al., 2012; Tassone et al., 2012). FXTAS occurs most frequently after age 50 in individuals who carry between 55 and 200 CGG repeats, also known as “premutation” carriers. More recently, cases of FXTAS have been identified in carriers of “gray zone” alleles (45–54 CGG repeats) (Hall et al., 2012). Clinically, FXTAS is defined by kinetic tremor, cerebellar ataxia, cognitive decline, psychiatric difficulties, mild parkinsonism, peripheral neuropathy, and autonomic dysfunction (Jacquemont et al., 2003, 2004; Louis et al., 2006; Berry-Kravis et al., 2007a,b; Leehey, 2009). Some women premutation carriers develop early infertility, a disorder referred to as Fragile-X associated primary ovarian insufficiency (Allingham-Hawkins et al., 1999).

Fragile-X associated tremor/ataxia syndrome has distinct features on MRI including severe generalized atrophy, cerebellar atrophy, and sub-cortical and/or ponto-cerebellar white mater lesions (Greco et al., 2006). More than half of men with FXTAS have an “MCP sign,” or T2 hyperintensity in the MCPs (Adams et al., 2007). Apartis et al. (2012) and Hall et al. (2016) also described hyperintensity in the splenium of the corpus callosum. This is the first reported case of a patient with FXTAS who presented to the ED with acutely worsening ataxia and was found to have an acute infarct in medial aspect of the left MCP (as well as a chronic infarct in the lateral aspect of the left MCP).

## Discussion

While the “MCP sign” is common in patients with FXTAS, to our knowledge this is the first reported case of an infarct in this location in a patient with this underlying neurodegenerative condition. Isolated MCP infarcts are rare (representing less than 0.15% of acute strokes) and are likely due to hypoperfusion in the watershed region between the anterior inferior cerebellar artery and the superior cerebellar artery (John et al., 2013). Pathologic studies in FXTAS have demonstrated spongiosis and axonal spheroids in the MCP as well as a range of mild to severe Purkinje cell loss and gliosis (Greco et al., 2006). We hypothesize that these damaged MCP fibers from FXTAS were at increased risk for ischemia, and the combination of small vessel disease due to poorly controlled vascular risk factors and a narrow left V4 segment of the vertebral artery may have led to transient hypo-perfusion during abrupt neck manipulation and preferential damage to these “at-risk” cerebellar Purkinje cells.

## Concluding Remarks

This case report suggests that FXTAS patients should have aggressive stroke risk factor management and that acute onset or worsening of cerebellar signs in these patients should be evaluated emergently.

## Ethics Statement

Written informed consent was obtained from the patient publication of this case report.

## Author Contributions

DH and RD: patient management and manuscript editing. AF: patient management, manuscript drafting and editing.

## Conflict of Interest Statement

The authors declare that the research was conducted in the absence of any commercial or financial relationships that could be construed as a potential conflict of interest. The reviewer VMC and handling Editor declared their shared affiliation.
